# Is 99m Tc-methylene diphosphonate bone scintigraphy a sensitive method for detecting bone lesions in multiple myeloma?

**DOI:** 10.22088/cjim.9.2.140

**Published:** 2018

**Authors:** Mohsen Vakili Sadeghi, Sadegh Sedaghat

**Affiliations:** 1 Cancer Research Center, Health Research Institute, Babol University of Medical Sciences, Babol, Iran.; 2 Department of Internal Medicine, Clinical Research Development Unit of Ayatollah Rouhani Hospital Babol University of Medical Sciences, Babol, Iran.

**Keywords:** 99m-Tc methylene diphosphonate, Bone lesion, Multiple myeloma

## Abstract

**Background:**

Bone lesion in multiple myeloma (MM) is most commonly presented as a lytic lesion in this disease. Determination of extent of bone lesions in MM is necessary to follow-up the patients. Whole body bone scan with 99m, Tc-methylene diphosphonate (MDP) has a lower sensitivity than other modalities.

**Methods:**

From the patients with MM admitted to Ayatollah Rouhani Hospital of Babol-Iran from 2009 to 2015, who had undergone whole body bone scan during diagnostic process, were entered into the study. Findings of bone scan were compared with MRI.

**Results:**

Of the 19 patients, sixteen (84.2%) of them had positive finding in bone scan, fifteen (78.9%) had MRI of the spine. While of the thirteen patients who had positive finding in MRI, seven (53.8%) had more positive finding in thorcolumbosacral MRI than in bone scan.

**Conclusions:**

99m-Tc MDP bone scan is a sensitive but insufficient method for detecting bone lesions in MM.

Multiple myeloma (MM) is a malignant disease of plasma cell due to its clonal proliferation. Bone lesion is a diagnostic criteria for MM and may present as lytic lesion, expansile mass lesion and/or osteopenia. Determining the extent of bone lesions in MM is necessary to follow-up the patients. Plain radiography, magnetic resonance imaging (MRI), positron-emission tomography/computed tomography (PET/CT) and Tc99m sestamibi (MIBI) scan have reasonable sensitivity for this purpose. Malignant plasma cells produce osteoclast activating factors (OAF), includingIL-1,IL-3 pathway,lymphotoxin, VEGF, tumor necrosis factor, macrophage inhibitory factor (MIP)-1 alpha/ᵦ, and receptor activator of NF-kappa B (RANK) ligand. Osteoblast activity is suppressed in MM with dickhoff-1 (DKK-1) produced by myeloma cells (1, 2). As a result, for detecting osteolytic bone lesions, plain radiography is better than isotopic bone scintigraphy (bone scan) (2), but it seems scintigraphy has a reasonable sensitivity. In this article, we reviewed the findings of 99m-Tc MDP bone scintigraphy in our MM patients. 

## Methods

The patients with MM admitted to Ayatollah Rouhani Hospital of Babol from 2009 to 2015 were evaluated; those who had undergone whole body bone scan during diagnostic process, entered into the study. Diagnosis of MM was done based on the last criteria of International Multiple Myeloma Working Group:1-equal or more than 10% plasma cells in bone marrow examination, 2- presence of monoclonal gammopathy in serum or urine and 3-an end organ damage (hemoglobin>2gr/dL below the lower limit of normal value or less than 10gr/dL, creatinine clearance <40 ml/min or creatinine> 2mg/dl, one or more lytic bone lesions and hypercalcemia) (3).

Bone lesions in thorcolumbosacral MRI reported with different radiologists were compared with bone scan findings. Whole body bone scan was performed with TC99 methylene diphosphonate (MDP) in different centers. The patients ‘data were analyzed with SPSS Version 22. 

## Results

Sixty-seven patients were evaluated from 2009 to 2015 and 19 patients entered the study. Thirteen (68.4%) of the patients were males and six (31.6%) were females. Their mean age was 59.16±12.75 years. 18 (94.7%) patients had bone pain at presentation. Table 1 demonstrates some data of the patients.

**Table 1:** Some charactristics of studied population

**Variable**	**Minimum**	**Maximum**	**Mean(±SD)**
Age(year)	39	81	59.16±12
Hemoglobin(gr/dL)	3.6	12.4	9±2.05
Platelet(/μL)	51000	335000	205±80
White blood cell(/μL)	3500	15300	7376±3642
ESR(1 hour)	12	130	83±31

Of the six women, one had hemoglobin level of 10.8gr/dl and another one with hemoglobin level of 12.4gr/dl at presentation and four patients had anemia. Of the 11 men, all had anemia, as a result, 88.3% of the patients had anemia at presentation. Other abnormal laboratory findings are demonstrated in table 2. 

**Table 2:** Frequency of some abnormal laboratory finding of the patients

**Variable**	**N (%)**
Leucopenia(<4000/µL)	3(15.8)
Anemia	18(94.7)
Thrombocytopenia(<150000/µL)	5(21.1)
Hypercalcemia(>1mg/dL higher than the upper limit of normal)	2(10.5)
Renal failure(creatinine>1.2mg/dL)	6(31.6)
Increased ESR	17(89.5)
Hypoalbuminemia(<3.5gr/dL)	8(42.1)

Of the 19 patients, sixteen (84.2%) had positive finding in bone scan. Fifteen (78.9%) patients had MRI of the spine. Two patients had negative finding not only in the bone scan but also in the MRI. Of the thirteen patients who had positive finding in MRI, seven (53.8%) patients had more positive finding in thorcolumbosacral MRI than in bone scan; certainly heterogeneity of bone marrow was seen in these cases in MRI. Of the 19 patients, seventeen (89.5%) had skull x-ray. Only ten (52.6%) had one or more punched- out lesions. 

## Discussion

The extent of bone lesions in MM can be determined with several methods. Plain x-ray, whole-body x-ray (WBXR) survey, is a conventional method and includes chest x-ray, skull x-ray, humeri, femoral bones, pelvic, cervical, thorasic and lumbosacral spine. But x-ray becomes positive when 50-70% of bone is lost, and has low sensitivity to detect early lytic lesions. Whole body MRI is another and more sensitive method (1, 2). 

Recently (18) fluorine-fluorodeoxyglucose ((18) F-FDG) positron-emission tomography/computed tomography (PET/CT) has been confirmed as a sensitive (80-90%) and specific method (90-100%) (4-6). Durie-Salmon plus classification MRI and FDG-PET/CT scan, are both recommended7. In Durie-Salmon plus classification, MRI and FDG-PET/CT scan are both recommended (7). MRI is more sensitive for detecting bone marrow infiltration with malignant plasma cells than PET/CT scan (4). Whole-body low dose CT is a new method, and may replace x-ray (8).Bone scintigraphy is not recommended for the assessment of bone lesions in MM via medicine textbooks but interesting results exist in the literature (9). 

Weng et al. in a systematic review of scintigraphy (Tc99m Sestamibi), MRI, FDG-PET and PET/CT for diagnosis of bone lesions in MM find equal sensitivity for the detection of osteolytic lesions between these procedures, with pooled sensitivity and specificity of 0.98 and 0.90, 0.88 and 0.68, 0.99 and 0.69, respectively. Furthermore, for bone scan, they were 0.66 and 0.83 (10).

Whole body Tc99m sestamibi was compared to Tc99m MDP by Alexandrakis et al. Sestamibi scan detects bone lesions in 78.5% of the 28 patients with MM and Tc99m MDP detects 53.5% (11). These values were 100% and 75%, respectively from 20 patients in the study of Alper E et al. (12). In another study, whole body sestamibi scan was very sensitive for evaluating the extent of bone lesions in MM (13). When amyloidosis complicates MM, Tc99m MDP scintigraphy can detect amyloid deposition besides bone lesions (14, 15). In one study, sensitivity and accuracy for the detection of bone metastasis was 85.7% and 70.1%, for bone scan and 97.1% and 92.3% for FDG-PET/CT (16). In another study, sensitivity and specificity for detection of bone metastasis of head and neck cancer were 88% and 98% for bone scan and 100% for PET/CT (17).

In our study, TC99m MDP bone scan was positive in 84.2% of the cases that is more sensitive than previous studies. Although in 53.8% of the cases, MRI is better for the detection of the extent of thoracolumbosacral spine lesions but bone scan is more feasible for the detection of bone lesions in the ribs and the extremity bones. PET/CT scan is available only in Tehran and is very expensive. With practical standpoint, we cannot perform neither whole body MRI nor PET/CT scan and Tc99m sestamibi scan is costly and not available extensively, as a result, we can perform whole body Tc99m MDP bone scan besides skull x-ray, chest x-ray and cervical thoracolumbosacral MRI for the detection and follow-up of bone lesions.

The important limitations of this study were the different centers for bone scan and different radiologists for MRI reporting. We suggest a prospective study in which bone scan and MRI be done for all patients in a single center. In conclusion, we showed that whole body Tc99m MDP bone scan has reasonable sensitivity for the detection of bone lesions in MM and can be used as a modality for this purpose in addition to other feasible methods. 
